# Assessment of interactive acoustic deterrent devices set on trammel nets to reduce dolphin–fishery interactions in the Northern Tyrrhenian Sea

**DOI:** 10.1038/s41598-023-46836-z

**Published:** 2023-11-24

**Authors:** Ilaria Ceciarini, Enrica Franchi, Francesca Capanni, Guia Consales, Lorenzo Minoia, Stefania Ancora, Antonella D’Agostino, Alessandro Lucchetti, Daniel Li Veli, Letizia Marsili

**Affiliations:** 1 Department of Physical Sciences, Earth and Environment, University of Siena, Via Pier Andrea Mattioli 4, 53100 Siena, Italy; 2 La Casa dei Pesci Onlus, Via Montianese 41, Fonteblanda, 58015 Talamone, Grosseto, Italy; 3 Department of Integrative Marine Ecology, Stazione Zoologica Anton Dohrn, Italian National Institute for Marine Biology, Ecology and Biotechnology, Genoa Marine Center (GMC), Via De Marini 6, 16149 Genoa, Italy; 4 Department of Business and Quantitative Studies, University of Naples Parthenope, Via Generale Parisi 13, 80132 Naples, Italy; 5 Institute for Biological Resources and Marine Biotechnologies (IRBIM), National Research Council (CNR), Largo Fiera della Pesca 1, 60125 Ancona, Italy; 6 Centro Interuniversitario per la Ricerca sui Cetacei (CIRCE), Department of Physical Sciences, Earth and Environment, University of Siena, Strada Laterina 8, 53100 Siena, Italy

**Keywords:** Ecology, Behavioural ecology, Biodiversity, Conservation biology

## Abstract

Dolphin–fishery interaction is a worldwide issue affecting dolphins through bycatch and fishers through catch or gear damages. Concerning the Mediterranean Sea, problematic interactions mainly occur between common bottlenose dolphin and small-scale fisheries. Acoustic Deterrent Devices such as pingers, are one of the most widespread measures used in attempts to face this issue. Therefore, the efficiency of interactive pingers (DiD01) in protecting the trammel nets from dolphin interactions was assessed in the Northern Tyrrhenian Sea. From March to October 2021, a total of 139 fishing trials using nets with pingers (TEST) and without pingers (CTRL), respectively *n* = 97 and *n* = 42, were carried out. Non-parametric statistic of the Catch per Unit Effort, comparing control and test nets, was not significantly different (*p* > 0.05) using catches weights (CPUE_W_) while it was significant (*p* < 0.01) considering the number of individuals (CPUE_N_). Moreover, richness and relative abundance of species resulted statistically higher in test nets (*p* < 0.05). This finding suggests that the absence of dolphin in the neighbourhood of fishing areas thanks to the use of pingers increases the diversity of target species. Catch damages caused by dolphins were statistically higher in nets without pinger than in nets with pinger (*p* < 0.05). No dolphin bycatch was recorded during fishing operations.

## Introduction

The Mediterranean Sea has always been the cradle of civilization, providing coastal populations with essential resources such as crustaceans, molluscs, and fish. Although it is considered a global hotspot of marine biodiversity^1,2^ this basin is subject to multiple and cumulative human-related activities that affect the marine environment, such as fishery^3,4^. Fisheries have long exerted such strong pressure that sustainable management is urgently needed to conserve marine ecosystems^5–7^. Commercial fishing activities often interact with non-target species, especially those that are more vulnerable, like sharks, marine turtles, as well as marine mammals such as seals and cetaceans^8–14^, and for this reason, these organisms are also frequently involved in the phenomenon of the incidental catch, also known as bycatch^15,16^. Nevertheless, fisheries give rise to a multitude of other impacts that affect the well-being of these species, e.g., noise pollution, behavioural changes, altered food availability, competitive pressures, chemical pollution, and population declines.

Among these major marine vertebrates, interactions between cetaceans and fishery are particularly significant, which is a worldwide and long-standing issue revealing, only in some parts of the world, positive consequences known as beneficial cooperation^17–24^. Actually, more often, this interaction negatively affects both mammalians (i.e., cetaceans and humans) that can be considered potential competitors for the same resources^25–34^. In the Mediterranean Sea, the conflicting relationship has been documented in several areas: Western Mediterranean Sea, Northern-Ionian Sea, Central Mediterranean Sea, Ligurian, and Aegean Sea occurring mainly between small-scale artisanal fishers and common bottlenose dolphins (*Tursiops truncatus*, Montagu, 1821)^35–47^. In addition to being a key species at the top of the food web, its opportunistic feeding behaviour, its coastal distribution home range, and marked adaptability, are all factors that inadvertently expose common bottlenose dolphin to fishing activities. These interactions cause not only direct effects such as injury and mortality, but also, provoke indirect impacts like feeding behavioural changes alteration of distribution, etc^48^. The harmful modalities through which common bottlenose dolphins interact include depredation, damage to catch and gear, and disturbance during fishing activities. For this reason, some fishers complain about dolphin presence and regard them as “enemies”. This anger led to the long-established practice of retaliatory culling which has persisted in recent years^49,50^, and fosters the illegal killing of dolphins for human consumption in some part of Italy (particularly in Liguria and Tuscany), where the dolphin fillet known as “musciame” can be still found in limited supplies on illegal markets^51,52^. Even if the sum of economic, ecological, and social damages directly correlated to dolphin–fishery interactions is hard to quantify and assess, it is widely recommended to seek resolutions through integrated and interdisciplinary approaches^33,53^. Three different approaches exist to mitigate harmful interactions between humans and wildlife: (1) to change human behaviour, (2) to modify the nature of the interaction and (3) the most challenging, to change the behaviour or distribution of animals without any changes in human behaviour^54^. Acoustic Deterrent Devices (ADD) such as pingers, are an example of the third approach and are one of the most widespread measures used in attempts to mitigate interactions between marine mammals and fishing gear^55^. Thanks to the extraordinary odontocete acoustic system, pingers are the best-known and most investigated tools in the reduction of common bottlenose dolphin depredation in scientific trials and commercial fishery^32,56–60^. Pingers are active sound emitters that produce a variety of acoustic signals from the middle to the high frequencies (10–180 kHz) at relatively low intensity (Sound Pressure Level—SPL < 180 dB re 1 μPa at 1 m). Different types of pingers were widely used, and formal results as well as fishers’ opinions, have been assessed for each type^61,62^. Though several studies have proven dolphin net damages and catch depredation reduction using ADD^54,59^, some experiments have shown that the constant sound emitted by the pinger generates dolphin habituation^63–65^. In these circumstances, pingers go from being deterrents to attractants, acting as a kind of “dinner bell” for dolphins. Interactive pingers, devices emitting high-frequency random and alarming sounds, should not induce dolphin habituation because they are activated by dolphin whistles or clicks, which are normally emitted for communication or predation purposes^66^. Nevertheless, the effectiveness of interactive pingers in reducing dolphin–fishery interactions, dolphins’ bycatch, and economic losses owing to the reduced quantity and/or quality of catches and damage to fishing gear is still under investigation^62^.

In 2021 common bottlenose dolphin Mediterranean subpopulation was reclassified by IUCN from Vulnerable^67^ to Least Concern, hopefully as a result of the effective management and conservation measures put in place. However, as harmful interaction with fisheries is not a ceased threat for its conservation, this topic needs to be further explored in order to find a solution and to prevent common bottlenose dolphin from being reclassified into higher-risk categories, as has occurred in the past^68^.

Within the framework of the Life DELFI project (LIFE18 NAT/IT/000942), 8 months of experimental trials with interactive pingers were conducted in the waters off the Southern Tuscan coast in 2021. This study was intended to: (**1**) test Dolphin Interactive Deterrents (DiD01), an interactive pinger, on trammel nets to keep common bottlenose dolphin far from fishing activities; (**2**) evaluate differences in terms of catch per unit effort per weight (CPUE_W_) and number of individuals (CPUE_N_); (**3**) evaluate differences in terms of richness and occurrence of species caught by nets with pingers and without pingers; and (**4**) evaluate the effectiveness and the efficiency of DiD01 in reducing harmful interactions such as the occurrence of catch and net damages.

## Study area, materials and methods

**Figure 1:**
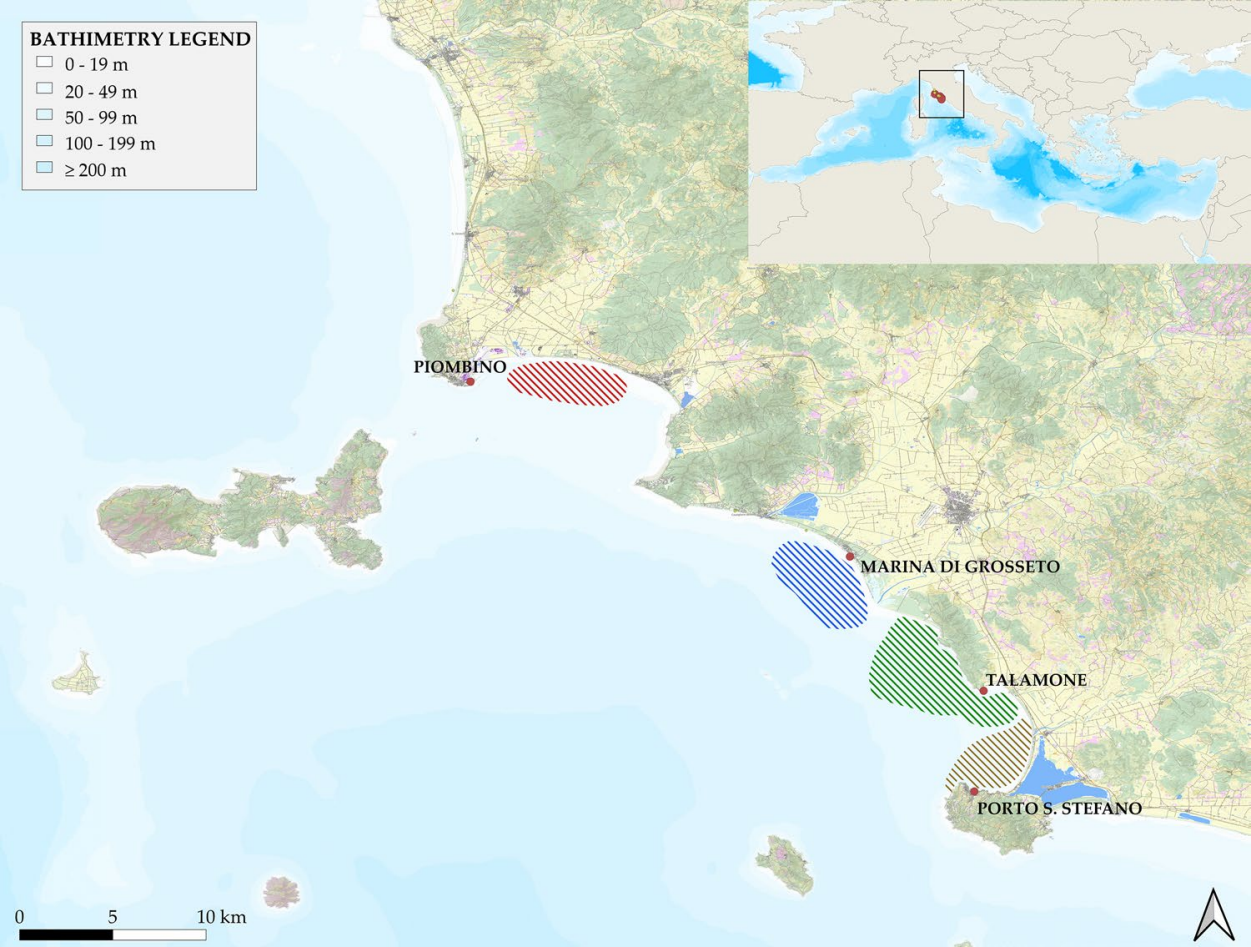
Study area map showing the four harbours and related study areas in the Northern Tyrrhenian Sea (Italy): “brown” AREA 1 (Porto Santo Stefano, GR), “green” AREA 2 (Talamone, GR), “blue” AREA 3 (Marina di Grosseto, GR) and “red” AREA 4 (Piombino, LI). Bathymetry legend shows the depth in various shades of colour from the lightest to the darkest. Map created with QGIS software version 3.28.0 “Firenze” (http://qgis.org).

Trials were conducted between March and October 2021 in nearshore waters along the southern Tuscan coast in the Northern Tyrrhenian Sea (Fig. 1). The study area is located within the Pelagos Sanctuary where the common bottlenose dolphin normally lives, as confirmed by the latest IUCN (2022) report^68^. In this study area, four fishing harbours with a majority of vessels of Small-Scale Fisheries (SSF) operating mainly in areas overlapping with common bottlenose dolphin distribution (as reported by fishers interviewed during Life DELFI Action C1 and D1 project bottom-up surveys^69^) were selected. The fishing areas were named: AREA 1 for Porto Santo Stefano (GR), AREA 2 for Talamone (GR), AREA 3 for Marina di Grosseto (GR) and AREA 4 for Piombino (LI). The whole study area covers around 112 km of coastline from Porto Santo Stefano (GR) to Piombino (LI). All fishing areas were within 12 miles off the coast, as expected by D.M. 12/7/2016, Art.1. Italian Regulation regarding the Italian SSF policy. In each area fishers with Small Scale Fisheries vessels interested in testing pingers were selected.

### Dolphin interactive Deterrents (DiD01) set up

**Figure 2:**
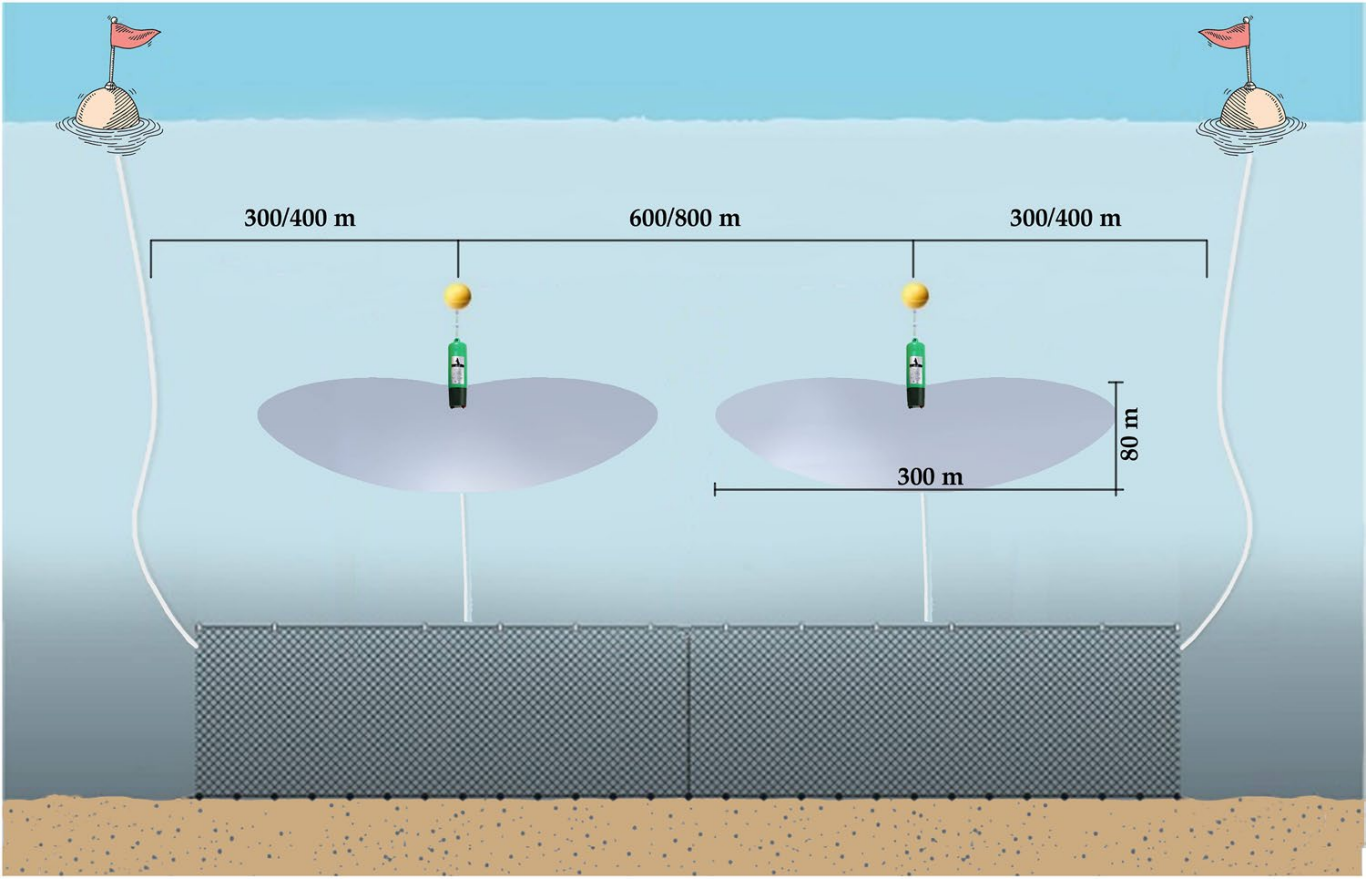
The setup of the DiD01 on a trammel net shows the correct distance between the buoyant signal and each pinger. The emission-range of the pinger is also shown in grey.

DiD01 acoustic deterrent devices used in the trials (STM Products S.r.l., Verona, Italy), are specifically designed to emit signals only in response to dolphin echolocation clicks. This implies that the pinger is typically in a standby or hearing mode and only activated when an integrated hydrophone detects clicks originating from a dolphin. The output signals are emitted from 5 up to 500 kHz at 168 dB re 1 μPa at 1 m as random high-speed tones FM ranging from 100 μs up to seconds (Table 1). The emission radius of a single device is 250 m, thus covering an horizontal space of 500 m, and extends 80 m downward, with an approximately toroidal emission field, as shown in Fig. 2 and well-explained in the user manual (Cod 2629006—Rev. 04.03—12.10.2020). The user manual provided by the manufacturer contain the following warning: “do not place a DiD01 at less than 600–800 m from another DiD01”. In addition, the technical features of the DiD is given in another section of the manual and confirmed that the horizontal spacing between two devices must be 600–800 m. To further maximizes the acoustic coverage, the first device is placed approximately 300 m from the start of the nets.

**Table 1:** DiD01 technical sheet (STM Products S.r.l., Verona, Italy).

Technical features	
Emission frequency	From 5 to 500 kHz
Emission power	168 dB re 1 μPa at 1 m
Maximum reception capability	125 dB re 1 μPa at 1 m in the 50–70 kHz
Maximum reception distance	800–1200 m with echolocation pulses > 200 dB
Minimum operative depth	10—20 m
Maximum operative depth	200 m
Horizontal spacing	600–800 m
Power internal source	5 rechargeable 1.2 NiMH batteries
Batteries autonomy	> 300 h in hearing mode. ~ 12 h in continuous emission mode
Average lifetime	500–1000 battery charge/discharge cycles
Dimension	210 × 61 mm
Weight	990 g

The pingers were deployed on the net using floating branch lines whose length varies according to the depth of the study area. The branching lines are made of polyethylene ropes (*Ø* = 8 mm), with two stainless steel snaps fasteners at both ends, to easily connect them to the net and the float. The latter is needed to neutralize the weight of the pinger (990 g). PVC deep water buoy (*Ø* = 400 mm; net buoyancy = 2 kg) was used to provide positive buoyancy. An additional lead weight (1 kg) was placed on the guideline for each branch, to prevent the net from detaching from the seabed due to water currents. Finally, the pinger was connected to the buoy by a snap. The setup of DiD01 on the trammel net is shown in Fig. 2.

### Data collection

All data were collected from both fishers and researchers in two different logbooks called respectively: “fisher logbook” and “observer logbook” shown in the Supplementary Material (Figure S1). A properly qualified observer of the Department of Physical Sciences, Earth, and Environment, (University of Siena) collected the data whenever it was possible to get on board. Otherwise, when, for some reasons, such as technical issues or weather conditions, there was not the possibility to get onboard, data was collected directly from the fishers. Then, after being initially checked by researchers, the data obtained via fishers’ logbook was then incorporated into the database using a data entry programme. Data were collected during the normal course of fishing operations, without interference from the scientists on board.

Experimental trials were conducted using trammel nets, usually set during sunset, and hauled just before or after sunrise, as in commercial fishing activities. Trials were divided into nets without pingers called “CTRL” and nets fitted with pingers called “TEST”, and so, in order to minimize differences due to patchy species distribution, the two types of nets were deployed, when possible simultaneously, close to each other maintaining the distance between TEST and CTRL net of 1000 m. Net meshes and height were equal between CTRL and TEST nets, while net length, reported in Table S1, differed between them.

At the beginning of the trial, the geographical coordinates of the net position, date and time, net feature (mesh size mm, length m, and height m), water depth m, and number of pingers employed for test nets were recorded. At the end of the trial, the date and time, the weight and number of specimens, and the scientific name of fish species caught using FAO code according to the ASFIS List of Species for Fishery Statistic Purposes 2022 version^70^ were recorded.

During all the fishing trials, the occurrence of dolphin was visually assessed during the hauling and setting phases and indirectly, it was inferred from the presence or absence of damage to the nets or catch caused by dolphins.

Economic loss due to damage to gear (number and size of holes) was estimated directly by fishers based on cost and time requested for repairing nets. Moreover, the economic loss due to the catch damages was calculated considering the potential economic value of the damaged fraction. These data were recorded in logbooks and throughout photos in order to assess if the damages were due to dolphin interaction or other causes. Only damages due to dolphin interaction were considered for the economic loss evaluation.

Maps of study area and TEST and CTRL trial distribution maps were made by using QGIS program 3.28.0 version. All pictures of fishing trials and fishers were taken during the study after obtaining a written informed consent from all human subjects. All subjects agreed to be photographed during fishing operations and gave their informed consent to have their identifying details (including photos) published.

### Statistical analyses

To compare CTRL and TEST net results, catch data were first standardized since the total length and the soak time^[^calculated as (DD/MM/YYYY, hh:mm *hauling time*)—(DD/MM/YYYY, hh:mm *setting time*)] between CTRL and TEST nets were different. Catches from each fishing trial were standardized using CPUE as in Lucchetti et al. (2019)^71^. CPUE in terms of weight (CPUE_W_) and in terms of number of individuals (CPUE_N_) were calculated as follows:

$${\text{CPUE}}_{{\text{W}}} = {\text{ W}}_{{\text{c}}} /\left[ {\left( {{\text{NetLength}}/{1}000{\text{ m}}} \right) \, \left( {{\text{NetSoakTime}}/{\text{12 h}}} \right)} \right]$$

$${\text{CPUE}}_{{\text{N}}} = {\text{N}}_{{\text{c}}} /\left[ {\left( {{\text{NetLength}}/{1}000{\text{ m}}} \right) \, \left( {{\text{NetSoakTime}}/{\text{12 h}}} \right)} \right]$$

where N_c_ and W_c_ are respectively the total number and total weight of captured individuals.

Above all, the catch per unit effort (CPUE) method was used to standardize the data and minimize any possible discrepancies in catch performance in terms of weight (kg) and the number of individuals (*n*), in order to overcome potential biases that could affect all trials such as weather conditions, dolphin behaviour, net disparities, and pinger activity.

Boxplots, barplots, and pie graphs were then used to display graphically results. Descriptive statistics were used to summarize the main characteristics of the control and test trials. The Mann–Whitney U non-parametric test was implemented to detect differences between CTRL and TEST CPUE_W_ and CPUE_N_, while the association between the presence of damages and CTRL vs TEST nets was explored using the Pearson Chi^2^ test^72^.

The species richness, i.e., the absolute number of species recorded in CTRL and TEST nets, has been estimated and explored graphically. Sørensen index (Sc) was used to assess the species similarity between the CTRL and TEST nets^73^. The equation for Sc is as follows:

$$Sc = \frac{2c}{{S_{CTRL} + S_{TEST} }}$$

where *c* is the number of species common to both net captures and $S_{CTRL}$ and $S_{TEST}$ are the number of species captured by CTRL and TEST nets, respectively.

This index takes values from 0 to 1: the closer to 1 the value is, the more similar the CTRL and TEST nets captures are.

Shannon Equitability Index (*E*_*H*_) was used to measure the evenness of species in CTRL and TEST nets’ captures^74^. Evenness refers to how similar the abundances of different species are in the CTRL and TEST net captures.

The Shannon Equitability Index (*E*_*H*_) is given by:

$$E_{H} = \frac{{ - \mathop \sum \nolimits_{i = 1}^{S} p_{i} lnp_{i} }}{{{\text{ln}}\left( S \right)}}$$

where $p_{i}$ is the relative abundance of one species on the total number of individuals captured, *ln* is the natural log, and *S* is the number of species. This index ranges from 0 to 1 where 1 indicates complete evenness. Statistical analysis was carried out with STATA^75^.

### Institutional review board statement

The study was conducted in accordance with the Life DELFI project procedures and approved by the CNR coordinator partner of the financed project.

## Results

### Control and test trials outcomes

Six vessels and ten fishers were involved in the trials. Vessels operating in the four different harbours from the southern to the northern part were: one vessel in AREA 1, two vessels in AREA 2, two vessels in AREA 3, and one vessel in AREA 4.

A total of 139 fishing trials were carried out from March 1st to October 10th, 2021. The total was 42 CTRL and 97 TEST trials. In Table 2 information regarding all trials carried out in each area are reported. Most trials were conducted in AREA 2 (*n* = 66) followed by AREA 3 (*n* = 33), AREA 4 (*n* = 22) and AREA 1 (*n* = 18).

**Table 2:** Number of trials conducted in each area along the southern Tuscan coast between March and October 2021 divided between control (CTRL) and test (TEST) trials and type of data collection (fisher or observer logbook).

DATA	AREA 1	AREA 2	AREA 3	AREA 4	TOT
CTRL fisher logbook	1	6	8	10	25
CTRL observer logbook	1	13	3	0	17
Tot. CTRL trials	2	19	11	10	42
TEST fisher logbook	8	17	12	12	49
TEST observer logbook	8	30	10	0	48
Tot. TEST trials	16	47	22	12	97
TOT. TRIALS	18	66	33	22	139
Vessels	1	2	2	1	6
Fishers	2	3	2	2	9
Period	May-Oct	Mar-Jul	May-Sep	Jun-Oct	Mar-Oct

The number of vessels, fishers employed, and experimental period are also shown. Abbreviations: Mar = March, Jun = June, Jul = July, Sep = September, Oct = October.

**Figure 3:**
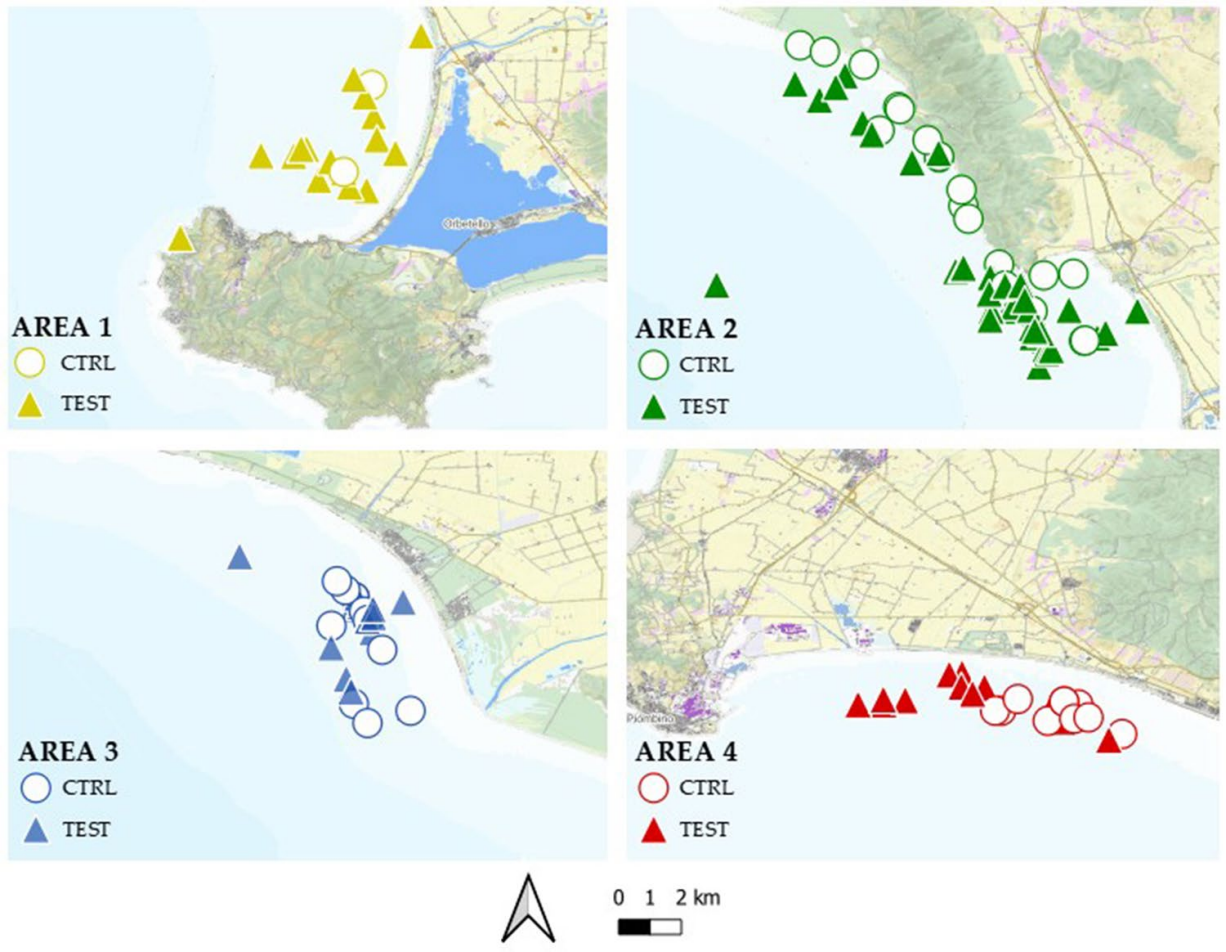
Map of the study area representing both CTRL (○) and TEST (▲) trials for AREA 1 in “yellow”, AREA 2 in “green”, AREA 3 in “blue” and AREA 4 in “red”. Map created with QGIS software version 3.28.0 “Firenze” (http://qgis.org).

Observer logbooks were not available in AREA 4 because it was impossible for researchers to get on board (boarding was not allowed since the boat lacked authorization to accommodate more than two individuals as per safety regulations, and the onboard staff already consisted of two fishermen). Figure 3 showed the locations where CTRL and TEST trials were carried out.

The technical features of the trammel nets varied depending on the study area. In AREA 1 the net height was 1.7 m, and the inner mesh size was 43 mm, while in AREA 2 they were, respectively, 1.5 m and 40 mm, in AREA 3 1.5 m and 45 mm, and in AREA 4 1.7 m and 45 mm. The average soak time and net length were shorter for CTRL nets than for TEST nets across all locations. The average water depth for control trials was 13.46 m, whereas it was 18.37 m for test trials, indicating that the fishers frequently fished in shallow seas close to shore. Further details on soak time (min), net length (m) and operating water depth (m) for CTRL and TEST nets in each area are shown in Supplementary Material (Table S1).

All species captured during the study, identified by fishers or observers during the trials, are reported in Table 3.

**Table 3:** List of species captured during experimental trials conducted between March and October 2021 in nearshore waters along the southern Tuscan coast.

Code	Scientific name & author	Common english name	Code	Scientific name & author	Common english name
SHELLFISH			GFB	*Phycis blennoides* (Brünnich 1768)	Greater forkbeard
Molluscs			HKE	*Merluccius merluccius* (Linnaeus 1758)	European hake
CTC	*Sepia officinalis* Linnaeus 1758	Common cuttlefish	HMM	*Trachurus mediterraneus* (Steindachner 1868)	Mediterranean horse mackerel
OCC	*Octopus vulgaris* Cuvier 1797	Common octopus	LDB	*Lepidorhombus boscii* (Risso 1810)	Four-spot megrim
SQF	*Loligo forbesii* Steenstrup 1856	Veined squid	LTA	*Euthynnus alletteratus* (Rafinesque 1810)	Little tunny (= Atl.black skipj)
Crustaceans			MGA	*Chelon auratus* (Risso 1810)	Golden grey mullet
			MLR	*Chelon labrosus* (Risso 1827)	Thicklip grey mullet
DPS	*Parapenaeus longirostris* (Lucas 1846)	Deep-water rose shrimp	MMH	*Muraena helena* Linnaeus 1758	Mediterranean moray
LBE	*Homarus gammarus* (Linnaeus 1758)	European lobster	MSF	*Arnoglossus laterna* (Walbaum 1792)	Mediterranean scaldfish
MTS	*Squilla mantis* (Linnaues 1758)	Spottail mantis squillid	MUF	*Mugil cephalus* Linnaeus 1758	Flathead grey mullet
SLO	*Palinurus elephas* (Fabricius 1787)	Common spiny lobster	MUR	*Mullus surmuletus* Linnaeus 1758	Surmullet
TGS	*Melicertus kerathurus* (Forsskål 1775)	Caramote prawn	MUT	*Mullus barbatus* Linnaeus 1758	Red mullet
FINFISH			MZZ	*Actinopterygii*	Marine fishes nei
Bony fishes			PAC	*Pagellus erythrinus* (Linnaeus 1758)	Common pandora
			SAA	*Sardinella aurita* Valenciennes 1847	Round sardinella
AMB	*Seriola dumerili* (Risso 1810)	Greater amberjack	SBG	*Sparus aurata* Linnaeus 1758	Gilthead seabream
ANK	*Lophius budegassa* Spinola 1807	Blackbellied angler	SBS	*Oblada melanura* (Linnaeus 1758)	Saddled seabream
ANN	*Diplodus annularis* (Linnaeus 1758)	Annular seabream	SLM	*Sarpa salpa* (Linnaeus 1758)	Salema
BBS	*Scorpaena porcus* Linnaeus 1758	Black scorpionfish	SNQ	*Scorpaena notata* Rafinesque 1810	Small red scorpionfish
BLL	*Scophthalmus rhombus* (Linnaeus 1758)	Brill	SOL	*Solea solea* (Linnaeus 1758)	Common sole
BLU	*Pomatomus saltatrix* (Linnaeus 1766)	Bluefish	SOS	*Pegusa lascaris* Ben-Tuvia 1990	Sand sole
BON	*Sarda sarda* (Bloch 1793)	Atlantic bonito	SSB	*Lithognathus mormyrus* (Linnaeus 1758)	Sand steenbras
BRF	*Helicolenus dactylopterus* (Delaroche 1809)	Blackbelly rosefish	SWA	*Diplodus sargus* (Valenciennes 1830)	White seabream
BSS	*Dicentrarchus labrax* (Linnaeus 1758)	European seabass	TUR	*Scophthalmus maximus* (Linnaeus 1758)	Turbot
CBM	*Sciaena umbra* Linnaeus 1758	Brown meagre	UUC	*Uranoscopus scaber* Linnaeus 1758	Stargazer
CBR	*Serranus cabrilla* (Linnaeus 1758)	Comber	WEG	*Trachinus draco* Linnaeus 1758	Greater weever
CEO	*Centrolophus niger* (Gmelin 1789)	Rudderfish	YRS	*Sphyraena sphyraena* (Linnaeus 1758)	European barracuda
COB	*Umbrina cirrosa* (Linnaeus 1758)	Shi drum	Cartilaginous fishes		
COE	*Conger conger* (Linnaeus 1758)	European conger			
CTB	*Diplodus vulgaris* (Geoffroy St. Hilaire 1817)	Common two-banded seabream	JRS	*Raja asterias* Delaroche 1809	Mediterranean starry ray
DEC	*Dentex dentex* (Linnaeus 1758)	Common dentex	RJC	*Raja clavata* Linneaus 1758	Thornback ray
EZS	*Scorpaena elongata* Cadenat 1943	Slender rockfish	RJO	*Dipturus oxyrinchus* (Linneaus 1758)	Longnosed skate
GPW	*Epinephelus aeneus* (Geoffroy St. Hilaire 1817)	White grouper	SDS	*Mustelus asterias* Cloquet 1821	Starry smooth-hound
GUU	*Chelidonichthys lucerna* (Linnaeus 1758)	Tub gurnard	TTV	*Torpedo torpedo* (Linnaeus 1758)	Common torpedo

Code, Scientific Name & Author, Common English name as expected by ASFIS List of Species for Fishery Statistics Purposes downloaded from 2022 version70 are also provided.

In Table S4 all species composing the common bottlenose dolphin diet^34^, together with the species collected by CTRL and TEST nets, were reported.

### Statistical validation of pinger efficiency

**Figure 4:**
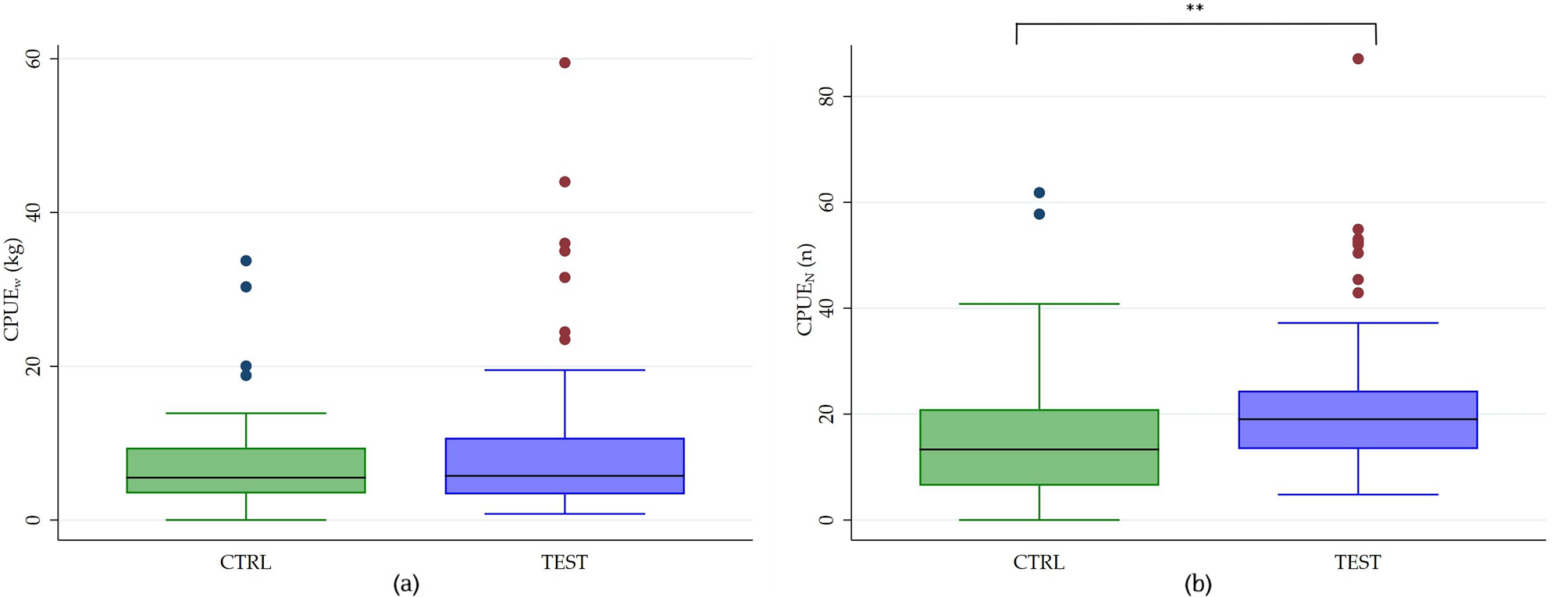
Boxplots of CPUE_W_ expressed in W_c_ /[(NetLength/1000 m) (NetSoakTime/12 h)] (**a**) and CPUE_N_ expressed in N_c_ /[(NetLength/1000 m) (NetSoakTime/12 h)] (**b**) calculated for CTRL and TEST trials in all four areas.

In Fig. 4, CPUE_W_ and CPUE_N_ values for CTRL and TEST trials are reported. The difference between CTRL and TEST nets was not significant in terms of CPUE_W_ (*z* = 0.795; *p* > 0.05) while it was statistically different for CPUE_N_ (*z* = 0.016; *p* < 0.01). In addition, CPUE_W_ and CPUE_N_ for each trial in each area are shown in Table S2.

### Effect of pinger on species composition

**Figure 5:**
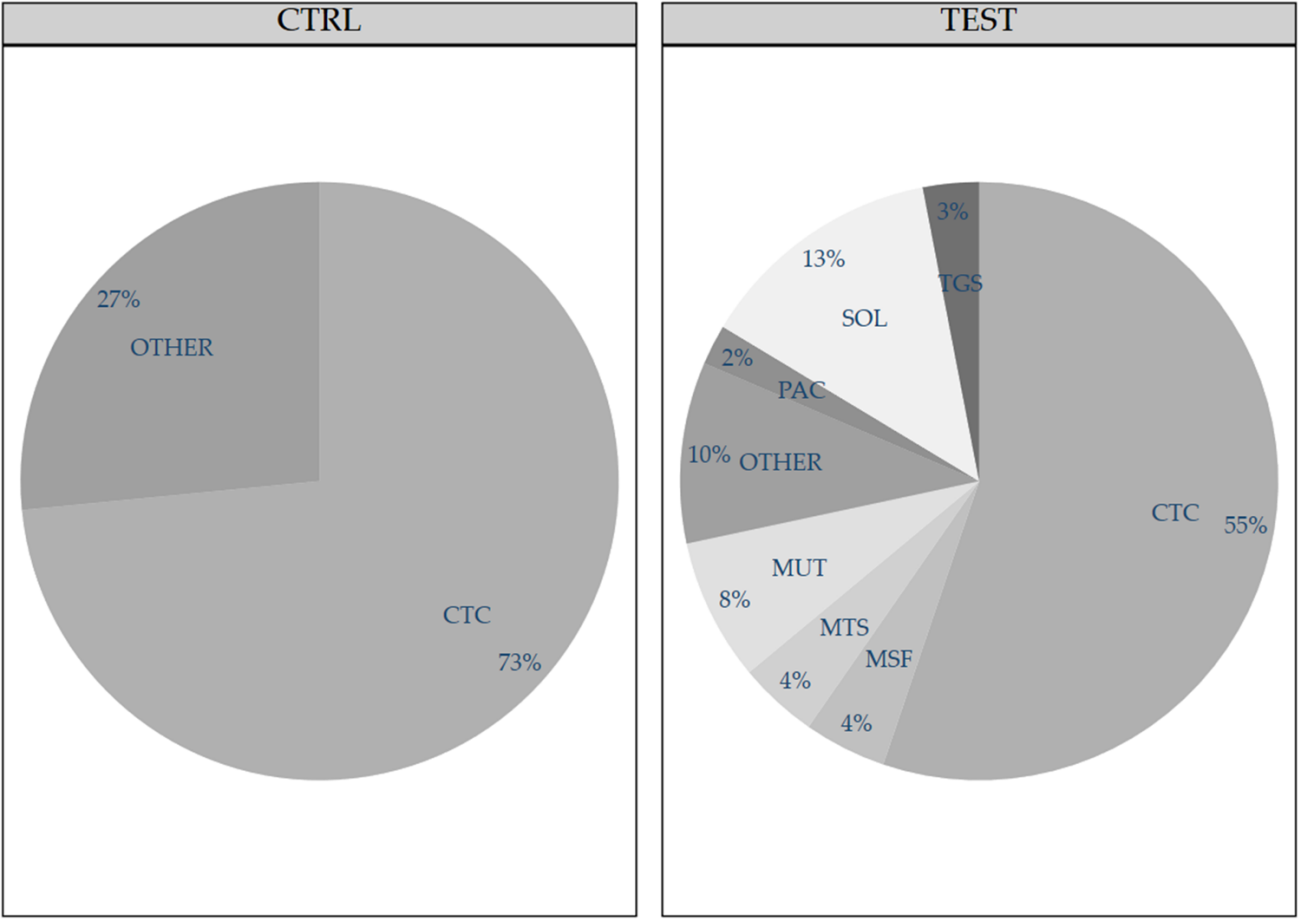
Relative abundance (%) of species caught in CTRL and TEST nets reported as FAO Code. “OTHER” includes species that present a number of individuals ≤ 100. The “OTHER” for both CTRL and TEST nets are shown in Supplementary Material (Table S3). The following acronyms correspond to: CTC = cuttlefish, MSF = Mediterranean scaldfish, MTS = spottail mantis squillid, MUT = red mullet, PAC = common pandora, SOL = common sole, TGS = caramote prawn.

Total species richness in all samples was 59, of which 15 species exclusively caught with TEST nets and 2 species were only present during CTRL trials. The cuttlefish (CTC) resulted the most relative abundant species representing 73% and 58% of total catches in the CTRL and TEST trials, respectively. In addition to the cuttlefish, in TEST nets other representative species caught were SOL accounting for 14% of total catches, red mullet (7%) and spottail mantis squillid and Mediterranean scaldfish (4% each) (Fig. 5).

The Sørensen index was 0.84, suggesting a change in species assemblages between CTRL and TEST nets.

The Shannon Equitability Index equal to 0.973 and 0.969 in CTRL and TEST nets, respectively, indicated a high degree of evenness between species in both catch composition/ CTRL and TEST net captures, with a slightly higher evenness in CTRL nets.

The occurrence (how many times) of species which were caught differed as it is shown in the graphics in the Supplementary Material (Figure S2). CTRL nets were able to capture 44 species of the 59 reported overall for CTRL and TEST trials. Among these, 21 species were only caught once (in only one area), 18 species were caught twice (in only two areas), 3 species (MUR, OCC, and SOL) were caught three times (in three areas), and only 2 species (CTC and MUT) were caught in all four areas. On the other side, TEST nets captured 57 species in total, with 25 species recorded once (only one area), 10 and 16 species were caught respectively twice (only two areas) and three times (in three areas), and 4 species (STS, MUT, SBG, and OCC) identified in all four areas.

### Effects of pingers on catch and/or net damages

During the whole trial, in 31.65% of the total 139 trials damages to the catches and/or to the net were recorded. Even if these 44 harmful interactions are equally shared between CTRL and TEST nets, they represent 52.38% for CTRL nets (*n* = 42) and 22.68% for TEST nets (*n* = 97).

Damages to the trammel net, identified as new holes in the inner net panel, were reported in a total of 24 trials. Overall, a total of 120 new holes were recorded in 19 CTRL and 4 TEST nets. Holes’ dimensions ranged from 30 to 200 cm, with an average of 78.61 ± 43.54 cm. Based on a previous study in which it was well explained the type of damage to catches if they were made by dolphins or other species^27^, it was possible to discriminate if the holes collected during the study were caused by dolphin interactions or other species interactions. In fact, among the total 24 trials, 18 trials reported damages from dolphins and 7 trials caused by other species (e.g., common octopus, sea fleas, moray eel or conger) or during fishing operations. Damages due by dolphin interactions were recorded 15 times in CTRL and 3 times in TEST trials. In Supplementary Material (Figure S3) morphological damages to the catches and to the gear collected during the study made by dolphin interactions and by other species were shown.

The highest economic loss related to catch damages occurred in AREA 4, accounting for a total of €139, and the lowest in AREA 2 where damages to the fishes did not even occur. On the other side, AREA 2 recorded the highest economic loss due to net damages amounting to €7650, followed by AREA 1 (€500), AREA 3 (€300), and AREA 4 (€85). Overall, the economic loss due to net damages amounted to €8535, much higher than the economic loss caused by catch damages, which was equal to €214. Statistical Pearson Chi^2^ test revealed a significant statistical association between the presence of damages in CTRL and TEST nets (Pearson chi2 (1) = 5.2297, Pr = 0.022).

### Effects of pingers on dolphin interactions

**Figure 6:**
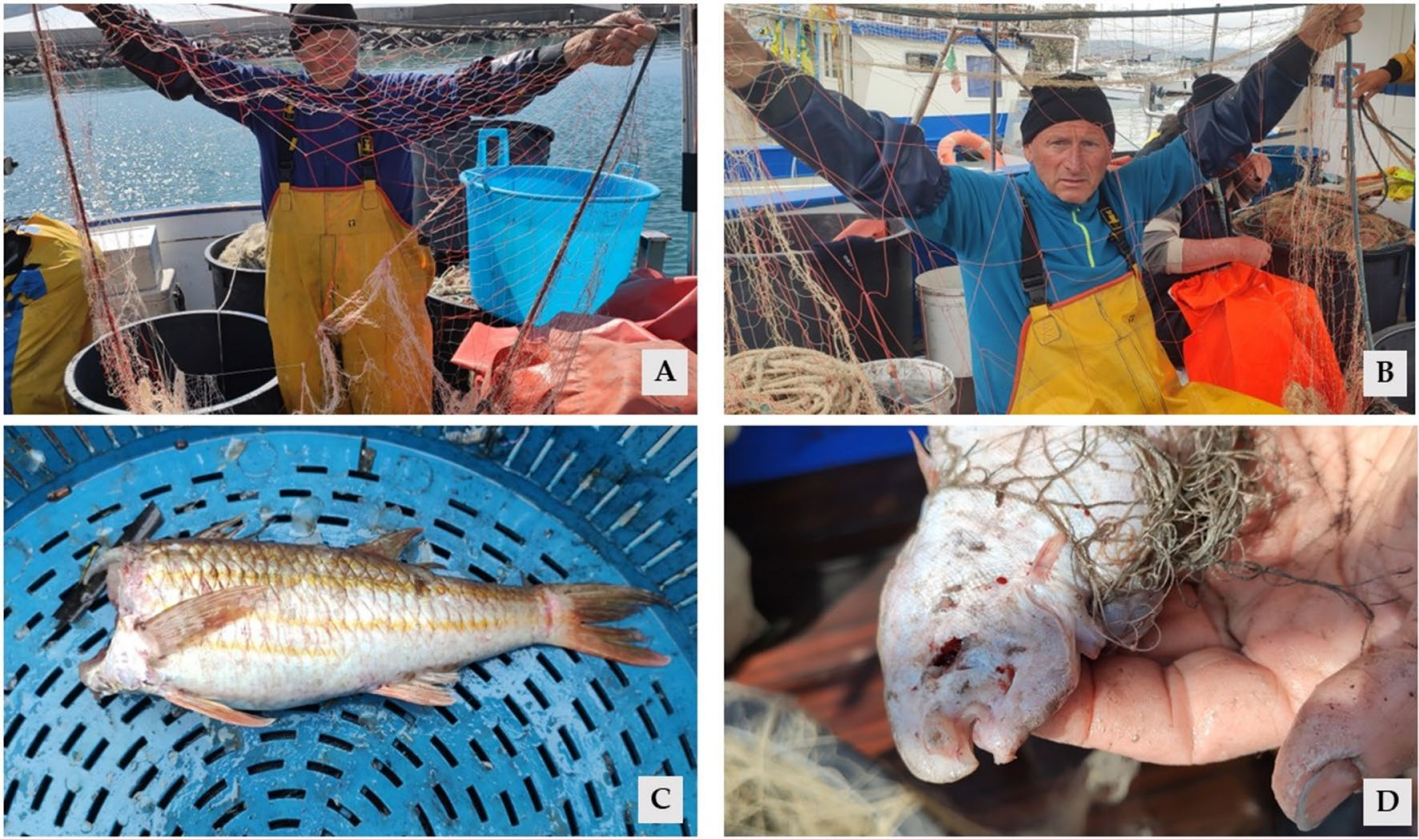
Fisher in AREA 2 shows damages caused by dolphins to the nets presenting holes in both inner and external panel of the trammel net (**A** and **B**). In this trial the also damages to the catch caused by dolphin bites occurred, for instance to the red mullet (**C**) and the common sole (**D**).

In Fig. 6 damages caused by dolphin both to the gear and to the catches are shown. During the study period, common bottlenose dolphin was the only cetacean species sighted and/or reported interacting with trammel nets.

Dolphin interactions (including sighting of harmful interactions) were recorded in 18 fishing operations, with the highest occurrence in AREA 4 (10 events), followed by AREA 2 (6 events), then AREA 1 and AREA 3 with each 1 event of interaction reported.

Among these 18 events of dolphin interactions, 16 involved CTRL nets and 2 TEST nets. The proportion of damages to the catches caused by dolphins was statistically different between CTRL and TEST nets (*z* = − 5.81, *p* = 0.000).

During hauling operations of 2 CTRL and 2 TEST nets, dolphins were seen close to the fishing gear. Three sightings were reported in July and one in October. In 3 of these 4 events, damage to catches and nets was documented. For instance, fishers working in AREA 1 reported a high number of holes (*n* = 22) in the gear as well as damage to the catch attributable to dolphins after observing 4 specimens during fishing operations with a CTRL net.

## Discussion

Our results show how difficult is to assess both the efficiency and the effectiveness of pingers in reducing dolphin–fishery interactions, while accounting for the fishers’ economic advantages or disadvantages. This is mostly due to the several variables to be considered, such as soaking time, water depth, geographic area, species richness, and the diet composition of the cetaceans, that could influence the frequency and, ultimately, the potential harmful effects, of dolphin–fishery interactions.

The present study proved DiD01 to be effective in reducing dolphin–fishery interaction without negatively affecting target and non-target catches. Indeed, CPUE_W_ in CTRL and TEST nets was not statistically significant. On the contrary, CPUE_N_ was significantly different in CRTL vs TEST nets, demonstrating that pingers do not affect the catches in terms of biomass (kg), but at the same time, TEST nets are able to catch more fish in terms of number of individuals. Furthermore, the highest species richness was reported for TEST nets, possibly—considering that other factors could be contributing to this result—due to the absence of dolphins in the neighbourhood of fishing grounds thanks to pinger activity.

Our results suggest that the use of pingers could increase the variability of target commercial species, avoiding, thus, the capture of only a few species. Nevertheless, the species richness and the total biomass of catches depended on a number of variables, such as fishing area, sea bottom features (muggy or rocky), fishing period, soak time, features of fishing gear, weather conditions, and many other environmental conditions. For example, target species such as cuttlefish are typically fished in near-shore waters at low water depths (< 15 m) in spring—which lasts from March 23rd until June 14th in the Mediterranean region^76^. During the summer, from June 15th to September 7th, the target species shift towards red mullet and other demersal species living in rocky habitats. Finally, between late summer and early autumn (from September 8th to December 2nd), fishing for medium-sized pelagic species was practiced.

It has been a long time since the scientific community started testing bycatch reduction devices, and during the last few decades, some studies have been conducted using acoustic deterrent devices in the Mediterranean Sea^32,41,57,58,62,77–80^. Although there are differences in the type of fishing gear, study area, fishing period, target species, and specific type of pingers used in all research studies in the literature, it is still possible to make comparisons in order to gain insights into the effectiveness and efficiency of pingers in reducing interactions between dolphins and fisheries.

First of all, some studies in the literature have documented a strong correlation between dolphin behavior and the availability of prey, which also happened to be the target species for small-scale fishers^27,28,42,45,81^.

In particular, stomach content analyses conducted on common bottlenose dolphin specimens stranded along the Tuscan coast^34^, found out two specimens with pieces of set nets in the stomach, confirming, so, the opportunistic feeding behaviour and the occurrence of fishing interactions with this cetacean species^34^ in the area. Moreover, the same research revealed that the diet composition of this species consists of 60 species belonging to Actinopterygii and Cephalopoda classes. With this in mind, the present study revealed a certain degree of overlap between the diet composition of the dolphins and the species captured with trammel nets in the experimental areas. Indeed, the 37.28% of the 59 species captured during the experimental trials, matched the species that were part of common bottlenose dolphin diet (i.e., ANN, BSS, CBR, COB, COE, CTB, DEC, GFB, GUU, HKE, HMM, MSF, MUF, MUT, OCC, PAC, SAA, SBG, SOL, SQF, SSB, and YRS). In particular 18 species were caught with both type of nets (CTRL and TEST), then three species included also in common bottlenose dolphin diet were caught solely using TEST nets. However, an important aspect that can be further investigated was the fact that cuttlefish, despite not being a target species for the common bottlenose dolphin diet in the Northern Tyrrhenian Sea, resulted the most frequently caught species in terms of the number of individuals (relative abundance). However, this was undoubtedly due to the fact that the study was based on the type of commercial fishing conducted by the involved fishers, who primarily target this resource typical of this marine environment.

The species caught by acoustic deterrents devices-equipped nets differed among studies according mainly to the fishing area and fishing gear. For example, in a study conducted in the Egadi Archipelago bottom gill nets were used with pinger model DDD2 by STM Products S.r.l. (0.1–150 kHz–160 dB re 1 μPa at 1 m) and catches were mainly composed by pelagic species such as bogue, round sardinella, and Atlantic horse mackerel^58^. Different results were found in the Aeolian Archipelago where trammel nets were used with Banana Pingers (5–120 kHz–145 dB re 1 μPa at 1 m), and the most captured species were parrot fish, forkbeard, and surmullet^62^. This result is in line with our study because, although cuttlefish and red mullet were the most abundant species captured, they are all demersal species. Interestingly, both studies, and also this study, showed that TEST nets’ catch compositions were more abundant in species that were part of common bottlenose dolphin diet (i.e., saddled seabream, common octopus, comber, *Phycis* sp. and *Pagellus* sp.).

Our findings revealed statistically significant differences in terms of CPUE_N_ between net equipped and not equipped with pingers, suggesting that pingers can increase the number of catches. Similarly, previous studies reported that net equipped with pingers prove to be more performing and favourable than net without pingers even if different variables such as gear features, seasonality, study area, pinger models and so on, were used^32,58,62,76^. Moreover, other papers focused on the study of the effect of pingers in the occurrence of some target species as herring, turbot, or Spanish mackerel, showing that acoustic deterrents did not negatively affect the catch of these target species^82–84^.

Comparisons regarding damages can be performed between the present study and an experiment conducted in the Balearic Islands where scientists used another type of pinger called Aquamark (20–160 kHz–145 dB re 1 μPa at 1 m) deployed on trammel nets for red mullet fishery^40^. In this case, damages to both catches and gears occurred more frequently in nets without pingers than in net equipped with pingers, as resulted in the present study with the 52.38% of CTRL nets with harmful interactions vs the 22.68% of TEST nets. Also, in Egadi Archipelago study, damages occurred in > 30% of control nets: a significantly higher number of small holes than the pinger net^56^.

Results about destructive interactions were similar, showing that damages, in terms of number of new holes caused certainly by dolphin interactions, were higher in nets without pingers than in nets equipped with pingers^58,85^. In a study conducted in the North of Cyprus, damages were six times greater^32^ and in another study carried out in Favignana Island there were more damages from dolphin control nets that in pinger-equipped net^58^.

The depredation phenomenon caused by dolphin interactions was discussed in some studies, for example one conducted in the Mediterranean Sea using DDD02 (STM Ltd.) pinger model obtaining a significant reduction of dolphin depredation; as similarly, the DiD01 model used in our study significantly minimizes dolphin interactions and their subsequent net-depredation^58,84^.

Also, two recent studies conducted one at sea using DDD02 (STM Ltd.) model and one along rivers using Future Oceans Inc. (10–70 kHz–132 dB re 1 μPa at 1 m) showed the good effectiveness of acoustic devices against dolphin entanglement^86,87^.

Negative experience using pingers were also reported^57,60,88^. In the Black Sea (Turkey), the Acquamark 100–200 (5–160 kHz–145 dB re 1 μPa at 1 m) model pingers did not reduce bycatch of harbour porpoise (*Phocena phocena*, Linneaus 1758)^60^. The same results were found for Fishtek Ltd., UK Banana pinger (59–130 kHz–145 dB re 1 μPa at 1 m) and the same species in the Kullaberg peninsula (North Sea, Sweden)^88^. Another study conducted in the Atlantic Ocean using SaveWave pingers (white model: 5–90, black model: 30–160 kHz–155 dB re 1 μPa at 1 m) proved dolphin habituation, confirming the “dinner bell” effect^57^. Instead, our study focused more closely on their effectiveness of this devices in fishing yield reducing dolphin–fishery interaction (see CPUE calculation), another concern about pinger use relates to the possible side effects caused by the increasing level of anthropogenic sound—specifically intended to deter cetaceans from an area, in an already very noisy environment. Particularly, if pinger use becomes widespread, the combined effect of a massive number of pingers might impact the physiology and auditory system of some cetaceans^89^. Whether acoustical devices produce some negative effects on dolphin hearing is still unclear^37^, since many factors can influence these potential side effects, such as duration of exposure, sound level, and spectral content^56^. In addition, some evidence may weaken the concept of pingers have a negative effect on the hearing of the bottlenose dolphin. However, some experiments on the characteristics of echolocation signals have shown that common bottlenose dolphin can emit echolocation signals with peak-to-peak 27 amplitudes as high as 225 dB re 1 μPa at 1 m^90^, which are much lower than those emitted by a common pinger (generally < 180 dB re 1 μPa at 1 m). Nevertheless, it is certainly worth considering for further research and studies the actual effectiveness of these tools, taking into account other factors, such as other side effects: long-term dolphin habituation, potential behavioural impacts on dolphins, and acoustic effects on other species in the marine environment.

## Conclusions

Overall, this study shows promising outcomes regarding the application of acoustic deterrent devices on trammel nets to reduce dolphin–fishery interactions. Although pingers have not yet been proven to fully resolve this issue for marine odontocetes, the DiD01 model (STM Products S.r.l.) did not negatively affect the abundance of catches, thus not causing economic losses to fishers, while increasing the species richness. Moreover, nets equipped with DiD01 did not record any cases of dolphin bycatch.

Our findings suggested that DiD01 could be attractive to fishers also because it did not require substantial changes to fishing operations or gear, and it did not require higher costs compared to alternative approaches.

Among the various technical mitigation methods proposed by Hamilton et al.^77^ used to reduce bycatch rates and marine mammals’ interactions with fishing operations, the use of acoustic deterrent devices was selected as the best one by the authors. The primary weakness of this technology lies in the risk of dolphin habituation to the sound emitted continuously and in the contribution to noise pollution given by this equipment’s. However, the DiD01 model is considered promising and innovative thanks to the random and irregular emission of sounds^91^.

Solving the problem of fishing interactions with dolphins to improve the economic performance of fishing activities, and, consequently, reduce losses, was a fundamental goal of this study. The other main objective was to preserve a cetacean species that, because of anthropogenic activities, is under pressure. In fact, it is worth keeping in mind that the common bottlenose dolphin is classified as an “ecologically relevant” species in the European Marine Strategy Framework Directive (MSFD, 2008/56/EC), it is considered a Species of Community Interest listed in Annex II of the Habitat Directive (Council Directive 92/43/EEC) and it is one of the most affected cetacean species by anthropogenic activities, because of its synanthropic behaviour. For this reason, in 2020 in the waters of the Northern Tyrrhenian and Ligurian Seas, the largest Mediterranean Site of Community Importance (SIC) (about 3740 km^2^) was established (Natura 2000 code IT516002—Regional Council Decision No. 2, of January 14th, 2020), specifically devoted to the conservation of this species.

In conclusion, this important issue could be only overcome with a multidisciplinary approach through: (**1**) regular monitoring of dolphin population in order to deepen the knowledge about its behaviour to direct conservation actions; (**2**) evaluate economic losses for fishers both due to gears and catches damages in order to foresee financial compensation measures as it has already been established in Sardinia Region (Italy) with a regional law (Reg.L. n. 19,824/Det/712 of 12/13/2018^92^); (**3**) manufacture new and more advanced pingers; (**4**) most importantly, raise awareness among fishers about the importance of their collaboration to manage and conserve the common bottlenose dolphin and the whole marine environment.

### Supplementary Information

Supplementary Information.

## Data Availability

All data generated or processed during this study is included in the manuscript and in the Supplementary information. Additional information about data availability can be requested to ilaria.ceciarini@student.unisi.it.
