# Supplementary material for: Assessment of interactive acoustic deterrent devices set on trammel nets to reduce dolphin–fishery interactions in the Northern Tyrrhenian Sea
Source: Sci Rep. 2023 Nov 24;13:20680. doi: 10.1038/s41598-023-46836-z (PMC10673957; doi:10.1038/s41598-023-46836-z)
Supplement: Supplementary file 1 — Supplementary Information. [file 41598_2023_46836_MOESM1_ESM.pdf]

Supplementary material

Assessment of interactive acoustic deterrent devices set on trammel nets to reduce dolphin-fishery interactions in the Northern Tyrrhenian Sea

Ilaria Ceciarini[1,2], Enrica Franchi[1], Francesca Capanni*[1], Guia Consales[1], Lorenzo Minoia[1,3], Stefania Ancora[1], Antonella D'Agostino[4], Alessandro Lucchetti[5], Daniel Li Veli[5], and Letizia Marsili[1,2,6]

[1] Department of Physical Sciences, Earth and Environment, University of Siena, Via Pier Andrea Mattioli 4, 53100 Siena, Italy
[2] La Casa dei Pesci Onlus, Via Montianese 41, Fonteblanda, Talamone, 58015 Grosseto, Italy
[3] Department of Integrative Marine Ecology, Stazione Zoologica Anton Dohrn, Italian National Institute for Marine Biology, Ecology and Biotechnology, Genoa Marine Center (GMC), Via De Marini 6, 16149 Genoa, Italy
[4] Department of Business and Quantitative Studies, University of Naples Parthenope, Via Generale Parisi 13, 80132 Naples, Italy
[5] Institute for Biological Resources and Marine Biotechnologies (IRBIM), National Research Council (CNR), Largo Fiera della Pesca 1, 60125 Ancona, Italy
[6] Centro Interuniversitario per la Ricerca sui Cetacei (CIRCE), Department of Physical Sciences, Earth and Environment, University of Siena, Strada Laterina 8, 53100 Siena, Italy

*Correspondence: francesca.capanni@unisi.it

SET NETS

OBSERVER LOGBOOK

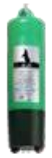

NET WITH PINGER ☐ NET WITHOUT PINGER ☐ HAUL N:

DEPTH (m):________________________________ VESSEL NAME:

FISHING AREA(Lat./Long.):___________________ NET TYPE (e.g. trammel):_________________________

NET LENGTH (m)____________ NET HEIGHT (n° meshes):______ MESH LENGTH (mm):__________

DATE AND SETTING TIME:__________________ DATE AND HAULING TIME:

NUMBER OF PINGERS:______________________ PINGERS SPACING (m):_____________________

Fill the table with the commercial species caught and details on non-marketable catch attributable to dolphin depredation. If an accurate measurement cannot be done, estimate the data.

COMMERCIAL SPECIES	Total number of individuals	Total weight (Kg)	N° non-marketable individuals	Total non-marketable catch weight (g)
Example: Common sole	*30*	*2.5*	*2*	*80 gr*
Number of dolphins sighted				
Distance between dolphins and nets				
Number of new damages (e.g. holes, tears)				
Diameter new holes (estimate in cm)				

Economic loss (estimate in €):	Catch damages	€______	Gear damages	€

Notes (e.g. dolphins' behaviour): ____

DISTRIBUTION SHEET

OBSERVER LOGBOOK

www.lifedelfi.eu

NET WITH PINGER ☐ NET WITHOUT PINGER ☐ HAUL N: ___________

DATE: ______ /________/_________ SPECIES: ________________________________

TOTAL WEIGHT:___________________ SUBSAMPLE WEIGHT: ___________________

LENGTH (cm)	NUMBER	LENGTH	NUMBER	LENGTH	NUMBER
1.5		18.5		35.5	
2		19		36	
2.5		19.5		36.5	
3		20		37	
3.5		20.5		37.5	
4		21		38	
4.5		21.5		38.5	
5		22		39	
5.5		22.5		39.5	
6		23		40	
6.5		23.5		40.5	
7		24		41	
7.5		24.5		41.5	
8		25		42	
8.5		25.5		42.5	
9		26		43	
9.5		26.5		43.5	
10		27		44	
10.5		27.5		44.5	
11		28		45	
11.5		28.5			
12		29			
12.5		29.5			
13		30			
13.5		30.5			
14		31			
14.5		31.5			
15		32			
15.5		32.5			
16		33			
16.5		33.5			
17		34			
17.5		34.5			
18		35			

Note: ____

Beneficiario coordinatore | Beneficiari associati

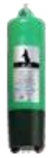

CETACEAN BYCATCH SHEET

OBSERVER LOGBOOK

NET WITH PINGER ☐ NET WITHOUT PINGER ☐

DATE: ______ /________/_________ SPECIES: ________________________________

INDIVIDUAL WEIGHT (Kg):_______________(measured/estimated)

TOTAL LENGTH (TBL): _______________(measured/estimated)

**Catch conditions**	Good	Bad	Inactive	Dead
**Release conditions**	Good	Bad	Inactive	Dead
**Sex**	M	F	IND.	
**ID image**				

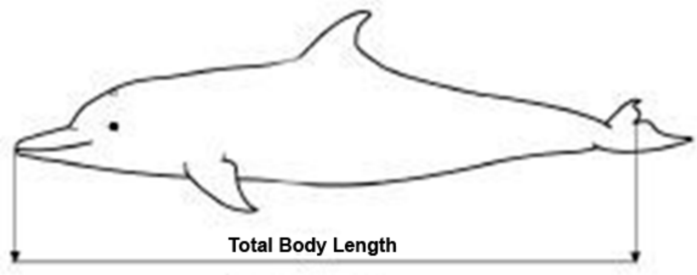

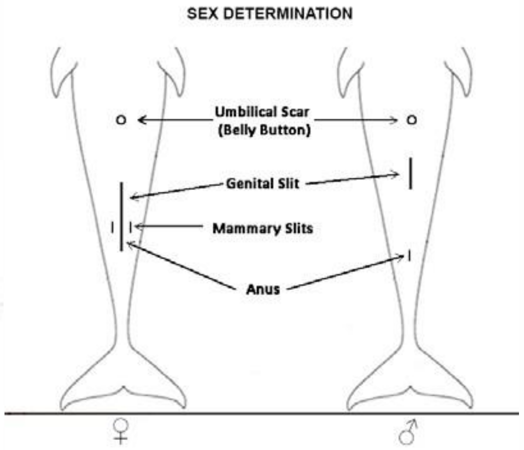

PRESENCE OF WOUNDS: ____

ADDITIONAL NOTES: ____

Other vulnerable species caught				
	**Species 1**	**Species 2**	**Species 3**	**Notes**
GROUP OF VULNERABLE SPECIES (Sea turtles, seabirds, sharks, rays and benthic species)				
SPECIES				
ID IMAGE				
NUMBER OF INDIVIDUALS				
TOTAL WEIGHT				
CATCH CONDITIONS				
RELEASE CONDITIONS				

SET NETS

FISHER LOGBOOK

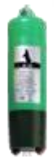

NET WITH PINGER ☐ NET WITHOUT PINGER ☐ HAUL N:

DEPTH (m):________________________________ VESSEL NAME:

FISHING AREA:____________________________ NET TYPE (e.g. trammel):_______________________

NET LENGTH (m)____________ NET HEIGHT (n° meshes):______ MESH LENGTH (mm):___________

DATE AND SETTING TIME:_________________ DATE AND HAULING TIME:

NUMBER OF PINGERS:_____________________ PINGERS SPACING (m):_____________________

Fill the table with the commercial species caught and details on non-marketable catch attributable to dolphin depredation. If an accurate measurement cannot be done, estimate the data.

COMMERCIAL SPECIES	Total number of individuals	Total weight (Kg)	N° non-marketable individuals	Total non-marketable catch weight (g)
Example: Common sole	*30*	*2.5*	*2*	*80 gr*

Number of dolphins sighted					
Distance between dolphins and nets					
Number of new damages (e.g. holes, tears)					
Diameter new holes (estimate in cm)					

Economic loss (estimate in €):	Catch damages	€______	Gear damages	€

Notes (e.g. dolphins' behaviour):____

Beneficiario coordinatore
Beneficiari associati

Action C1, D1

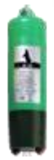

CETACEAN BYCATCH SHEET

FISHER LOGBOOK

NET WITH PINGER ☐ NET WITHOUT PINGER ☐

DATE: ______ /________/_________ SPECIES: ___________________________________

INDIVIDUAL WEIGHT (Kg):________________(measured/estimated)

TOTAL LENGTH (TBL): _______________(measured/estimated)

**Catch conditions**	Good	Bad	Inactive	Dead
**Release conditions**	Good	Bad	Inactive	Dead
**Sex**	M	F	IND.	
**ID image**				

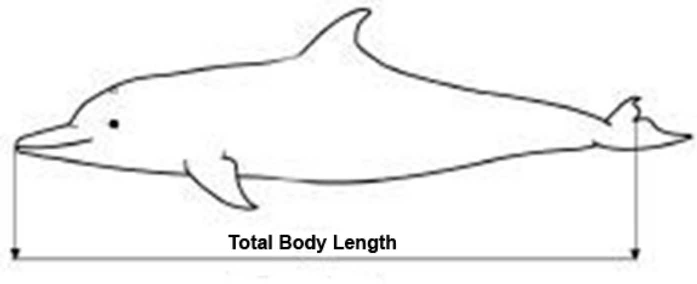

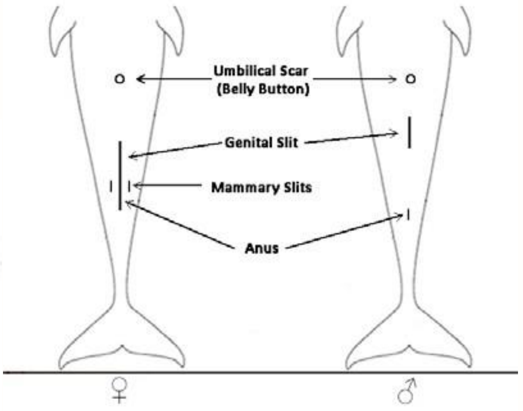

PRESENCE OF WOUNDS: ____

ADDITIONAL NOTES: ____

Other vulnerable species caught				
	**Species 1**	**Species 2**	**Species 3**	**Notes**
GROUP OF VULNERABLE SPECIES (Sea turtles, seabirds, sharks, rays and benthic species)				
SPECIES				
ID IMAGE				
NUMBER OF INDIVIDUALS				
TOTAL WEIGHT				
CATCH CONDITIONS				
RELEASE CONDITIONS				

Beneficiario coordinatore

Beneficiari associati

Figure S1. Observer and fisher’s logbook template.

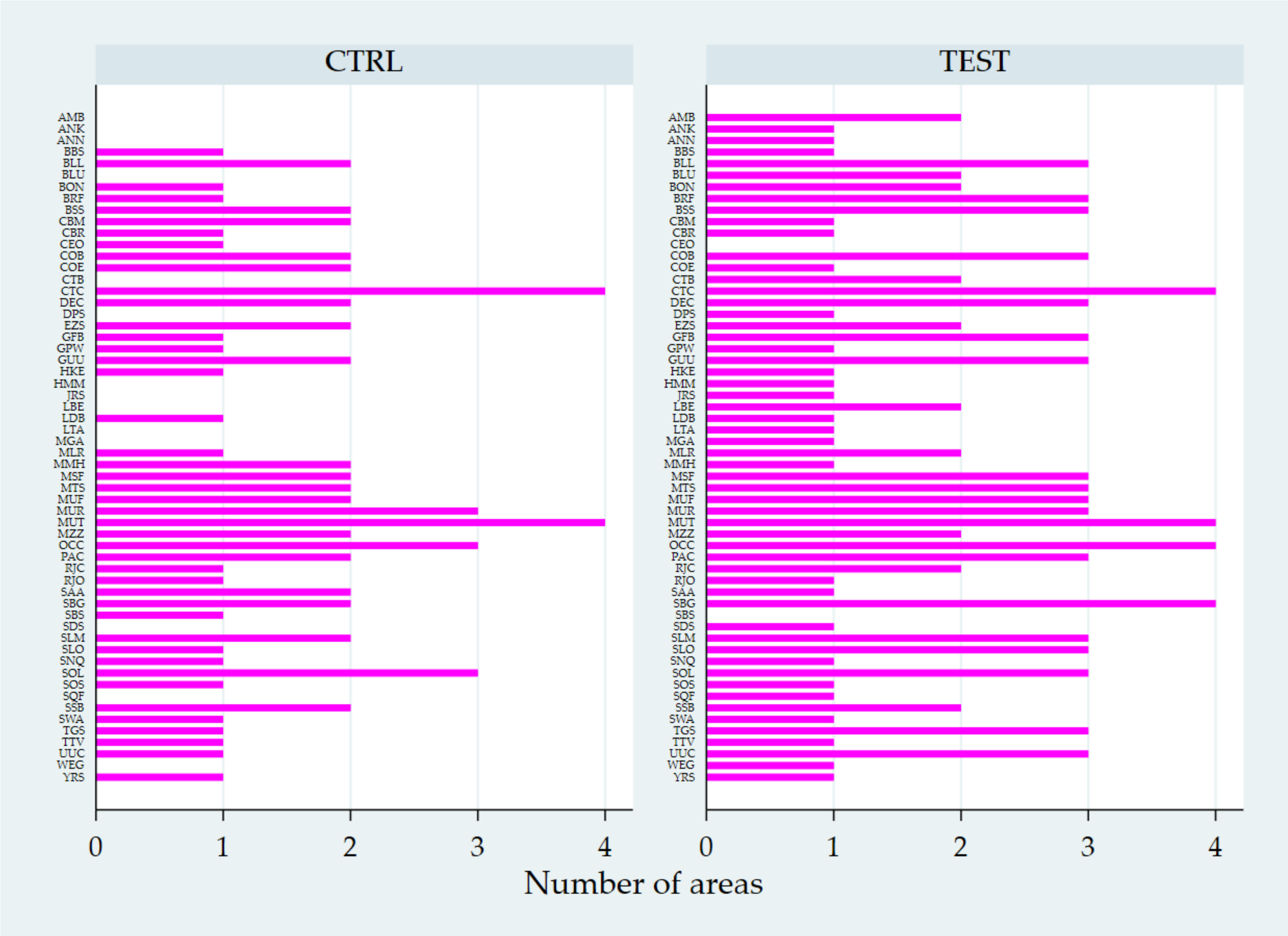

Figure S2. Times for each species caught between the four areas for both CTRL and TEST net. The species are reported with FAO Code. CTRL nets wereable to capture 44 species of the 59 reported overall for CTRL and TEST trials. Among these, 21 species were only caught once (only one area), 18 species were caught twice (only two areas), 3 species (MUR, OCC, and SOL) were caught in three times (in three areas), and only 2 species (CTC and MUT) were caught in all four areas. On the other side, TEST nets captured 57 species in total, with 25 species recorded in once (only one area), 10 and 16 species were caught respectivelytwice (only two areas) and three times (in three areas), and 4 species (STS, MUT, SBG, and OCC) identified in all four areas.

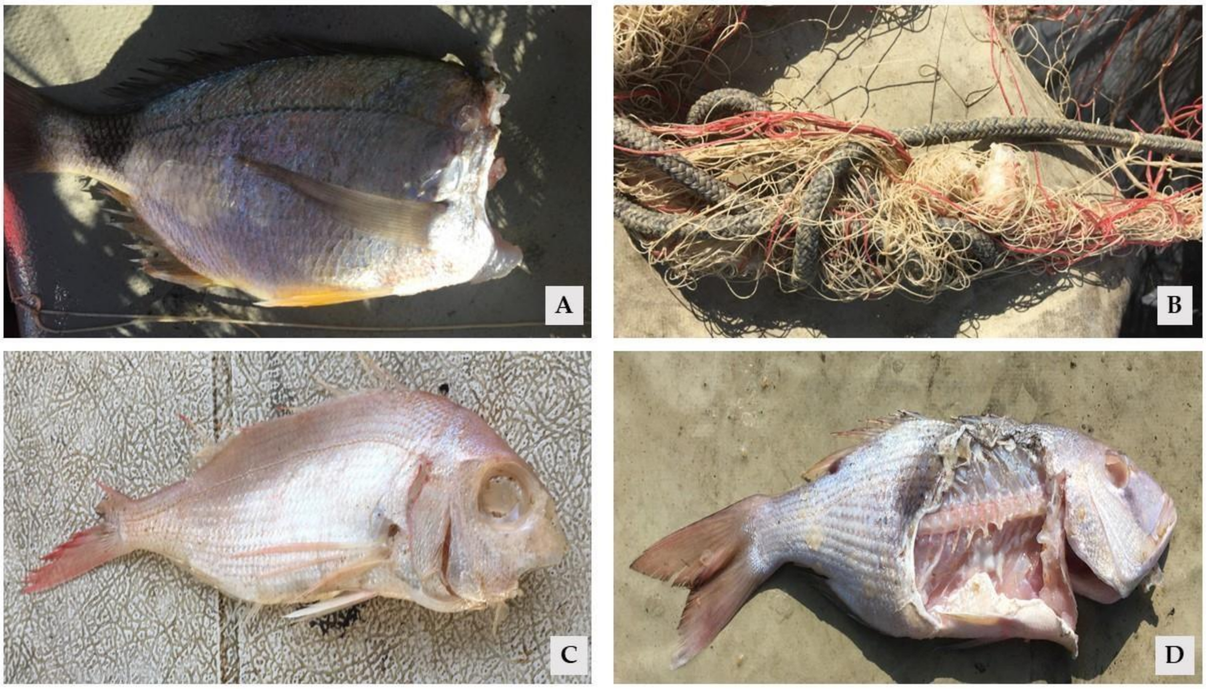

Figure S3. Damages to the catch: **A.** *Diplodus* sp. bitten by conger, **B.** Knot of conger, **C.** Common pandora scavenged by sea fleas and **D.** Gilthead seabream damaged by octopus' bite.

Table S1. Descriptive statistic values: "n" number of hauls, "Mean", minimum "Min" and maximum "Max" values and standard deviation "SD" of control and test hauls (CTRL and TEST) for soak time (min), net length (m) and water depth (m) in each area.

		**SOAK TIME (min)**		**NET LENGHT (m)**		**DEPTH (m)**	
**AREA**/TRIALS		CTRL	TEST	CTRL	TEST	CTRL	TEST
**AREA 1**	***n***	***2***	***16***	***2***	***16***	***2***	***16***
	Mean	1092.50	902.50	1050.00	1525.00	13.00	16.28
	Min	930	240	600	800	12	8
	Max	1255	1365	1500	2000	14	26
	SD	229.80	298.15	636.39	450.92	1.41	5.23
**AREA 2**	***n***	***19***	***47***	***19***	***47***	***19***	***47***
	Mean	1974.47	2423.21	1447.36	1606.38	9.99	20.79
	Min	780	1110	800	1000	2.5	4
	Max	2940	4290	2500	3000	20	28
	SD	822.77	732.90	415.48	392.54	6.01	6.27
**AREA 3**	***n***	***11***	***22***	***11***	***22***	***11***	***22***
	Mean	907.18	943.40	1312.72	1686.36	18.2	16.68
	Min	664	625	840	1000	13	12
	Max	1145	1200	2400	3200	31.5	31.5
	SD	182.55	212.29	454.95	684.72	8.03	6.88
**AREA 4**	***n***	***10***	***12***	***10***	***12***	***10***	***12***
	Mean	743.90	681.16	500.00	4583.33	14.95	14.79
	Min	510	510	500	4500	12	10
	Max	1579	1579	500	5000	21	21
	SD	317.58	285.25	0	194.62	2.77	3.65
**TOTAL AREAS**	***n***	***42***	***97***	***42***	***97***	***42***	***97***
	Mean	1359.95	1621.24	1167.62	1979.38	13.46	18.37
	Min	510	240	500	800	2.5	4
	Max	2940	4290	2500	5000	31.5	31.5
	SD	809.21	952.47	536.15	1087.33	6.74	6.39

Table S2. $CPUE_W$ expressed in W_c /[(NetLength/1000m) (NetSoakTime/12h)] and $CPUE_N$ expressed in N_c /[(NetLength/1000m) (NetSoakTime/12h)] values collected in every trial carried out using CTRL and TEST nets. Mean ± Standard Deviation are also reported.

**$CPUE_W$**								**$CPUE_N$**					
**CTRL**				**TEST**				**CTRL**			**TEST**		
**AREA1**	**AREA2**	**AREA3**	**AREA4**	**AREA1**	**AREA2**	**AREA3**	**AREA4**	**AREA1**	**AREA2**	**AREA3**	**AREA1**	**AREA2**	**AREA3**
9.41	5.02	20.04	4.80	15.89	8.39	19.04	4.80	7.55	17.16	20.01	6.48	20.71	15.47
10.32	6.75	33.73	8.58	8.46	4.54	24.47	13.29	11.11	13.42	16.92		15.40	11.53
	6.02	6.21	5.76	18.74	6.39	17.06	10.68	22.15	19.10	19.57		7.31	20.94
	3.40	4.58	11.19	1.82	13.33	36.00	8.57	18.70	8.18	8.11		9.69	40.81
	3.45	3.38	6.94	14.98	2.37	15.37	5.72	15.35	23.17	19.00		28.73	57.78
	8.15	13.26	30.33	17.24	3.71	34.99	11.73	6.25	21.29	13.40		16.69	61.83
	6.80	18.81	8.79	9.53	6.02	23.49	6.94	13.22	24.43	19.71		8.38	26.69
	2.23	5.25	13.89	1.44	11.59	10.69	16.80	13.22	10.61	29.83		25.56	9.14
	2.23	3.91	9.55	11.67	8.69	9.24	12.90		26.48	37.22		4.48	13.31
	1.41	11.02	7.20	19.50	6.44	10.60	9.67		22.81	30.85		4.96	14.79
	4.90	3.95		5.74	3.36	13.49	8.39		16.65	18.17		12.25	20.78
	3.46			3.25	2.31	5.51	9.16		19.45	87.11		22.55	
	5.16			4.24	7.92	15.57			9.97	42.91		11.05	
	0.00			59.48	5.79	9.43			20.36	54.90		0.00	
	3.02			31.56	4.55	7.20			19.79	53.06		4.64	
	2.84			44.00	4.22	5.76			24.89	8.68		4.93	
	4.36				3.05	5.29			23.30	52.00		4.58	
	0.34				3.58	4.51			23.44	27.69		14.69	
	4.00				1.65	1.83			7.90	20.73		2.75	
					2.80	5.09			50.41	18.47			
					1.09	3.01			52.52	36.88			
					2.27	3.17			9.71	35.59			
					3.35				45.41				
					5.13				30.80				
					5.07				20.71				
					3.62				22.35				
					4.95				20.99				
					2.30				13.49				
					8.31				17.45				
					2.97				15.13				
					3.92				5.87				
					1.39				12.94				
					3.27				18.67				
					3.12				4.80				
					2.40				14.17				
					3.63				19.03				
					1.74				14.98				
					4.26				18.54				
					4.23				21.05				
					4.23				6.61				
					4.64				5.82				
					2.01				15.43				
					0.80				5.00				
					1.44				5.56				
					6.21				29.29				
					7.98				15.66				
					1.86				34.93				
**Mean ± Standard Deviation**				**Mean ± Standard Deviation**				**Mean ± Standard Deviation**			**Mean ± Standard Deviation**		
9.87±0.6	3.87±2.1	11.28±9.5	10.70±7.3	16.72±16.0	4.40±2.6	12.76±9.79	9.89±3.4	13.44±5.33	19.14±10.7	18.32±18.7	/	11.55±8.25	26.64±18.5

Table S3. The other species indicated with FAO code that were caught from both CTRL and TEST nets with a number of individuals ≤100.

CTRL NET	TEST NET
BBS	AMB
BLL	ANK
BON	ANN
BRF	BBS
BSS	BLL
CBM	BLU
CBR	BON
CEO	BRF
COB	BSS
COE	CBM
DEC	CBR
EZS	COB
GFB	COE
GPW	CTB
GUU	DEC
HKE	DPS
LDB	EZS
MLR	GFB
MMH	GPW
MSF	GUU
MTS	HKE
MUF	HMM
MUR	JRS
MUT	LBE
MZZ	LDB
OCC	LTA
PAC	MGA
RJC	MLR
RJO	MMH
SAA	MUF
SBG	MUR
SBS	MZZ
SLM	OCC
SLO	RJC
SNQ	RJO
SOL	SAA
SOS	SBG
SSB	SDS
SWA	SLM
TGS	SLO
TTV	SNQ
UUC	SOS
YRS	SQF
	SSB
	SWA
	TTV
	TUR
	UUC
	WEG
	YRS

Table S4. Species list about common bottlenose dolphin diet species [34], CTRL/TEST nets, TEST and CTRL nets separately. In **bold** species matched between *Tt* diet and captures.

N.	***Tursiops truncatus* diet**	**CTRL/TEST nets**	**TEST nets**	**CTRL nets**
**1**	***Arnoglossus sp.***	*Actinopterygii*	***Diplodus annularis***	*Centrolophus niger*
**2**	*Abralia veranyi* (Rüppell, 1844)	***Arnoglossus laterna***	***Diplodus vulgaris***	*Oblada melanura*
**3**	*Alloteuthis* spp.	***Chelidonichthys lucerna***	*Euthynnus alletteratus*	-
**4**	*Ancistroteuthis lichtensteinii* (Férussac [in Férussac & d'Orbigny], 1835)	*Chelon labrosus*	*Homarus gammarus*	-
**5**	*Argonauta argo* Linneaus, 1758	***Conger conger***	*Chelon auratus*	-
**6**	*Ariosoma balearicum* (Delaroche, 1809)	***Dentex dentex***	***Loligo forbesii***	-
**7**	*Boops boops* (Linneaus, 1758)	***Dicentrarchus labrax***	*Lophius budegassa*	-
**8**	*Bothus podas* (Delaroche, 1809)	*Diplodus sargus*	*Mustelus asterias*	-
**9**	*Callionymus risso* Lesueur, 1814	*Dipturus oxyrinchus*	*Parapenaeus longirostris*	-
**10**	*Callionymus* sp. Linneaus, 1758	*Epinephelus aeneus*	*Pomatomus saltatrix*	-
**11**	*Cepola macrophthalma* (Linneaus, 1758)	*Helicolenus dactylopterus*	*Scophthalmus maximus*	-
**12**	*Chelidonichthys cuculus* (Linneaus, 1758)	*Lepidorhombus boscii*	*Raja asterias*	-
**13**	***Chelidonichthys lucerna*** (Linneaus, 1758)	***Lithognathus mormyrus***	*Seriola dumerili*	-
**14**	*Chelon ramada* (Risso, 1827)	*Melicertus kerathurus*	*Pegusa lascaris*	-
**15**	*Citharus linguatula* (Linneaus, 1758)	***Merluccius merluccius***	*Trachinus drago*	-
**16**	***Conger conger*** (Linneaus, 1758)	*Mugil cephalus*	***Trachurus mediterraneus***	-
**17**	***Dentex dentex*** (Linneaus, 1758)	***Mullus barbatus***	-	-
**18**	***Dicentrarchus labrax*** (Linneaus, 1758)	***Mullus surmuletus***	-	-
**19**	***Diplodus annularis*** (Linneaus, 1758)	*Muraena helena*	-	-
**20**	***Diplodus vulgaris*** (Geoffroy Saint-Hilaire, 1817)	***Octopus vulgaris***	-	-
**21**	*Eledone cirrhosa* (Lamarck, 1798)	***Pagellus erythrinus***	-	-
**22**	*Eledone moschata* (Lamarck, 1798)	*Palinurus elephas*	-	-
**23**	*Engraulis encrasicolus* (Linneaus, 1758)	***Phycis blennoides***	-	-
**24**	*Gnathophis mystax* (Delaroche, 1809)	*Raja clavata*	-	-
**25**	*Gobius niger* Linneaus, 1758 B	*Sarda sarda*	-	-
**26**	*Gobius* spp.	***Sardinella aurita***	-	-
**27**	*Heteroteuthis dispar* (Rüppell, 1844)	*Sarpa salpa*	-	-
**28**	*Histioteuthis reversa* (Verrill, 1880)	*Sciaena umbra*	-	-
**29**	*Illex coindetii* (Vérany, 1839)	*Scophthalmus rhombus*	-	-
**30**	*Lesueurigobius* sp.	*Scorpaena elongata*	-	-

N.	*Tursiops truncatus* diet	CTRL/TEST nets	TEST nets	CTRL nets
31	***Lithognathus mormyrus*** (Linneaus, 1758)	*Scorpaena notata*	-	-
32	**Loligo sp.**	*Scorpaena porcus*	-	-
33	*Loligo vulgaris* Lamarck, 1798	*Sepia officinalis*	-	-
34	***Merluccius merluccius*** (Linneaus, 1758)	***Serranus cabrilla***	-	-
35	*Micromesistius poutassou* (Risso, 1827)	***Solea solea***	-	-
36	***Mullus barbatus*** Linneaus, 1758	***Sparus aurata***	-	-
37	***Mullus*** **sp.**	***Sphyraena sphyraena***	-	-
38	***Octopus vulgaris*** Cuvier, 1797	*Squilla mantis*	-	-
39	*Onychoteuthis banksii* (Leach, 1817)	*Torpedo torpedo*	-	-
40	*Ophidion barbatum* Linneaus, 1758	***Umbrina cirrosa***	-	-
41	*Pagellus acarne* (Risso, 1827)	*Uranoscopus scaber*	-	-
42	***Pagellus erythrinus*** (Linneaus, 1758)	-	-	-
43	***Phycis*** **sp.**	-	-	-
44	*Pomadasys incisus* (Bowdich, 1825)	-	-	-
45	*Sardina pilchardus* (Walbaum, 1792)	-	-	-
46	***Sardinella aurita*** Valenciennes, 1847	-	-	-
47	***Serranus cabrilla*** (Linneaus, 1758)	-	-	-
48	Serranus sp.	-	-	-
49	***Solea solea*** (Linneaus, 1758)	-	-	-
50	**Solea sp.**	-	-	-
51	***Sparus aurata*** Linneaus, 1758	-	-	-
52	***Sphyraena sphyraena*** (Linneaus, 1758)	-	-	-
53	*Spicara flexuosa* Rafinesque, 1810	-	-	-
54	*Spicara smaris* (Linneaus, 1758)	-	-	-
55	*Spondyliosoma cantharus* (Linneaus, 1758)	-	-	-
56	*Synodus saurus* (Linneaus, 1758)	-	-	-
57	***Trachurus mediterraneus*** (Steindachner, 1868)	-	-	-
58	*Trachurus* sp.	-	-	-
59	*Trisopterus capelanus* (Lacepède, 1800)	-	-	-
60	***Umbrina cirrosa*** (Linneaus, 1758)	-	-	-
